# Hospital Statistics: Some Criticisms and Suggestions

**Published:** 1913-10-18

**Authors:** 


### HOSPITAL STATISTICS.

### Some Criticisms and Suggestions.

If one studies the train of events which has led up to the achievement of any great advance in the practice of medicine, it will usually be found that the analysis of a large number of individual observations has formed an important item. It has been observed that an "event" A has tended to be associated with or followed by an "event" B—that is to say, the two events have been *correlated.* Such correlation having been ascertained, its observer has been led to reflect upon the underlying causes of the association, and has arrived at important truths. Sydenham's improvements in the treatment of fevers and Semmelweis' great work in controlling puerperal septicæmia are cases in which the observer and discoverer were one and the same person. Again, the correlation of A and B may have long been known, but its significance have remained obscure until the advent of a specially gifted investigator. Modern advances in our knowledge of malaria and plague are instances of this. Finally, there are cases in which the correlation of the events A and B has neither been observed nor even suspected until an experimenter has suggested where one should look by advancing reasons for thinking that A and B would or should occur together. In other words, the association of A and B has sometimes been the starting-point of the inquiry, sometimes the last step, but—and this is the significant fact to note—at some stage in the inquiry its study has always been essential. It is, indeed, this fact which renders the collection and analysis of mass observations so important in medicine, and the science of statistics, which is merely the systematic treatment of such mass observations, a valuable instrument of medical research. Let it be asserted that a drug A is of benefit in a disease B; how can we learn whether the assertion is well founded? We shall probably be told that in cases of B treated with A the issue was favourable in a larger percentage than when A had not been used. The validity of the conclusion will depend in the first instance upon the magnitude of the difference and the extent of the experience. We shall need some process which will enable us to decide whether the difference is greater than could safely be set down as a chance event. We shall also need to inquire whether any factor other than the giving or not giving of A differed in the two sets of cases. Both these inquiries pre-suppose adequate statistical data and a methodical process of statistical analysis. This research, which is typical of most clinical inquiries, involves difficulties of two kinds: (1) the collection of data; (2) the analysis of data.

To take the second point first, it may be remarked that statistical analysis, like any other branch of science, is beset with pitfalls and demands some measure of technical skill. Until quite recently such technical skill was restricted to a small number of professional mathematicians, and instruction in the subject was difficult to obtain. At the present time facilities for obtaining instruction are ample. In London alone there are at least two statistical laboratories under the direction of specialists where courses are arranged to meet the needs of different classes of students, while to those unable to attend classes such excellent text-books as Mr. G. Udny Yule's recent "Introduction to the Theory of Statistics" will be of service, enabling the intelligent reader to gain an insight into the chief methods of the statistician, matters which were once thought to be a sealed book to all but those well acquainted with higher mathematics.

It will appear, therefore, that, so far as the actual methods of statistical analysis are concerned, the path of the medical statistician is reasonably smooth. With respect to the first point mentioned—the provision of data—the state of affairs is different. In December 1912 the Council of the Royal Statistical Society appointed a special committee with the following reference: "To consider the question of the morbidity and mortality statistics in the United Kingdom, and to report to the Council." When one remembers that the twelve metropolitan hospitals to which medical schools are attached together dispose of more than 5,000 beds, it might be expected that their records would have been of great service to the committee. The committee, however, reported on this point as follows: "Some of the larger hospitals publish tabulations of admissions to their wards, but none of them any of patients treated in their out-patient departments. A number of reports received by the committee have been examined, and only two have been found to contain tables showing the distribution of admissions by disease, sex, and age, distinguishing recoveries from fatal terminations." *

The present condition of hospital records may be illustrated by a personal experience. The writer desired to study the influence of age and sex upon the fatality of a common disease, and also to ascertain whether the fatality had changed during the last fifty years. For this purpose he had access to the records of a great hospital. Although the disease is among the chief causes of death and presents in most cases a characteristic clinical picture, the cases occurring each year were not separately tabulated, but had to be extracted individually from the admission book, a task which alone involved much labour when several years had to be included. If any more detailed information had been sought than the bare particulars of age, sex, occupation, and length of stay in hospital, it would have been necessary to refer to another series of volumes not separately indexed or arranged upon a nosological system. This absence of systematic tabulation offers an insuperable obstacle to anyone who requires statistical data from the hospitals within a short time. If, for instance, anyone wished to obtain a tabular statement of the cases of acute endocarditis in the wards of the London

* *Journ. Roy. Stat. Soc.,* LXXVI., 1913, p. 793.

hospitals during 1912, age, sex, occupation, duration of disease and termination being distinguished, its compilation would entail an immense amount of labour on the part either of voluntary workers or the hard-pressed officials of the hospitals. There is no uniform system of recording in force, neither are the officials trained in the work of preparing statistical returns and able to point out the fallacies and sources of error to which such returns are liable. In some of these respects the institutions under the Metropolitan Asylums Board compare favourably with the general hospitals.

There can be little doubt that this practical absence of available statistics, so far as the general hospitals are concerned, is a serious evil. To begin with, it is impossible to study the secular changes in fatality which may occur in the hospital population in the case of certain diseases. Also the absence of any large body of statistics which might serve as a control results in undue importance being attached to short series of cases extracted by some private worker. The danger of this is obvious when it is a question of deciding whether some particular line of treatment is or is not of advantage in a given disease. The inadequacy of the statistics which sometimes form the chief contents of articles in medical journals is only too apparent.

A still more important point is that the absence of adequate data renders a proper study of what we may term the "general hospital population" almost impossible. We know that this "population" is not a random sample, but a selection of the inhabitants of the area within which the hospital is situated; it is, indeed, a rather special selection. The consequence is that the fatality of disease within the wards of a hospital is very different from that prevailing outside. Everyone is more or less distinctly aware of this fact, but to attain clearness we require an accurate statistical study of the hospital population. It is most important that accurate knowledge of the special characters of a general hospital population should be readily available, not only for the light it throws upon such matters as the relation between poverty and disease, but also because the medical profession acquire their first and most vivid impressions of morbid phenomena within the walls of a hospital, and the danger of generalising from these impressions is considerable. The ideal is, of course, the codification of *all* medical experience, but so stupendous a task is not likely to be even contemplated until the relatively easier one—viz., a codification of hospital experience—has been achieved. It would be easy to adduce other reasons why a reform in the statistical systems of the general hospitals is required, but those cited will suffice to indicate the case in favour of a change.

In a second article it is proposed to sketch the changes which seem called for in the statistical organisation of the hospitals.

